# Western-type diet modulates inflammatory responses and impairs functional outcome following permanent middle cerebral artery occlusion in aged mice expressing the human apolipoprotein E4 allele

**DOI:** 10.1186/1742-2094-10-102

**Published:** 2013-08-20

**Authors:** Hiramani Dhungana, Taisia Rolova, Ekaterina Savchenko, Sara Wojciechowski, Kaisa Savolainen, Anna-Kaisa Ruotsalainen, Patrick M Sullivan, Jari Koistinaho, Tarja Malm

**Affiliations:** 1 Department of Neurobiology, A. I. Virtanen Institute for Molecular Sciences, Biocenter Kuopio, University of Eastern Finland, P.O. Box 1627, FI-70211 Kuopio, Finland; 2 Duke University and Geriatric Research Education Clinical Centre, DVAMC, Durham NC 27710, USA; 3 Department of Oncology, Kuopio University Hospital, P.O. Box 1627, FI-70211 Kuopio, Finland

**Keywords:** Brain ischemia, Inflammation, Apolipoprotein E, Atherosclerosis, Western diet, Aging

## Abstract

**Background:**

Numerous clinical trials in stroke have failed, most probably partially due to preclinical studies using young, healthy male rodents with little relevance to the heterogenic conditions of human stroke. Co-morbid conditions such as atherosclerosis and infections coupled with advanced age are known to contribute to increased risk of cerebrovascular diseases. Clinical and preclinical studies have shown that the E4 allele of human apolipoprotein (ApoE4) is linked to poorer outcome in various conditions of brain injury and neurodegeneration, including cerebral ischemia. Since ApoE is a known regulator of lipid homeostasis, we studied the impact of a high-cholesterol diet in aged mice in the context of relevant human ApoE isoforms on the outcome of focal brain ischemia.

**Methods:**

Aged mice expressing human E3 and E4 isoforms of ApoE in C57BL/6J background and C57BL/6J mice fed on either a high-fat diet or a normal diet underwent permanent middle cerebral artery occlusion. The impact of a high-cholesterol diet was assessed by measuring the serum cholesterol level and the infarction volume was determined by magnetic resonance imaging. Sensorimotor deficits were assessed using an adhesive removal test and the findings were correlated with inflammatory markers.

**Results:**

We show that expression of human ApoE4 renders aged mice fed with a western-type diet more susceptible to sensorimotor deficits upon stroke. These deficits are not associated with atherosclerosis but are accompanied with altered astroglial activation, neurogenesis, cyclooxygenase-2 immunoreactivity and increased plasma IL-6.

**Conclusions:**

Our results support the hypothesis that ApoE alleles modify the inflammatory responses in the brain and the periphery, thus contributing to altered functional outcome following stroke.

## Background

Cerebral ischemia is characterized by complex changes at both the molecular level and the cellular level in the affected brain parenchyma. Ischemic damage initiates an acute inflammatory response that serves to restore tissue homeostasis through clearance of cellular debris, neurogenesis and tissue repair, but the outcome is largely detrimental and contributes to delayed brain damage [1]. A number of anti-inflammatory compounds that have demonstrated neuroprotection in rodent models of brain ischemia have failed in human clinical trials [2]. Even though stroke affects older people with co-morbidities such as atherosclerosis, diabetes and even Alzheimer’s disease, the vast majority of the preclinical experiments are performed on homogeneous cohorts of young male rodents. This may have been a contributory factor to series of unsuccessful clinical trials testing neuroprotective compounds [2].

The primary function of apolipoprotein E (ApoE) is to maintain lipid homeostasis through the catabolism of triglyceride-rich lipid components [3]. ApoE is polymorphic and exists in three human isoforms (ApoE2, ApoE3 and ApoE4) that are encoded by the corresponding allele APOE*2, APOE*3 and APOE*4[4]. The most common ApoE allele (APOE*3) is considered the normal allele exhibiting a neutral risk phenotype in the majority of the population. APOE*4, however, is associated with increased risk of cardiovascular disease, stroke and Alzheimer’s disease [5,6] and is a risk factor for cognitive impairment in old age [7] and after stroke [4]. One of the mechanisms by which ApoE isoforms contribute to the outcome after central nervous system (CNS) insult is immunomodulation, as different ApoE isoforms have been shown to regulate both systemic and CNS inflammation [8].

Co-morbid conditions and risk factors for stroke such as atherosclerosis and obesity include a systemic inflammatory component and exacerbate the subsequent ischemic damage. In addition, aged mice show altered cytokine responses upon focal ischemia [9]. Even though ApoE has an essential role in lipoprotein homeostasis and has been shown to have a robust effect on the lesion size after ischemic insult [10-13], the impact of different ApoE isoforms on the outcome of inflammatory responses in aged mice on an atherogenic diet has not been assessed. In this study, we investigated the impact of a high-fat (HF) diet on sensorimotor functions and inflammation after permanent middle cerebral artery occlusion (pMCAo) in aged male mice expressing human E3 or E4 isoforms of human ApoE in C57BL/6j background, namely ApoE3 and ApoE4 targeted replacement (TR) mice. We hypothesized that the expression of human ApoE3 and ApoE4 isoforms together with long-term intake of a HF diet would differentially contribute to the outcome of focal cerebral ischemia and ischemia-induced inflammatory reactions. The HF diet used in this study represents a typical human cuisine in western countries, and since the majority of stroke patients are older we used aged mice. ApoE3-TR and ApoE4-TR mice offer a valuable tool for assessing the contribution of human ApoE isoforms in the outcome of stroke in the context of unhealthy human food intake and old age, thereby increasing the clinical relevance of the research compared with studies using naïve healthy mice.

## Methods

### Animals

All animal experiments were approved by the National Animal Experiment Board of Finland and followed the Council of Europe Legislation and Regulation for Animal Protection. ApoE3-TR mice and ApoE4-TR mice on a C57Bl/6J background (8X generation backcross) were obtained from Duke University Medical Center, Durham, North Carolina, USA (Patrik M Sullivan) and maintained in the Lab Animal Center, Kuopio, University of Eastern Finland. ApoE3-TR and ApoE4-TR mice were created as described [14]. Wild-type (WT) mice of C57BL/6J background were used as controls. The animals were housed in conventional facilities individually caged in a light and humidity controlled environment with chow and water available *ad libitum*. Thirteen-month-old male mice were randomized into treatment groups using Graph Pad QuickCalcs (GraphPad Software, Inc., La Jolla, CA, USA). Mice from each genotype – WT (*n* = 79), ApoE3-TR (*n* = 56) and ApoE4-TR (*n* = 77) – were divided into three subgroups: ischemic mice fed with a high-fat diet (HF + Isch), ischemic mice fed with a normal diet (ND + Isch) and sham-operated mice fed with a normal diet (ND + Sham). The western-type HF diet (Teklad TD88137 containing 48.5% carbohydrates, 21.2% fat and 0.2% cholesterol with energy density of 4.5 kcal/g; Harlan Laboratories, Madison, WI, USA) or the ND (Teklad 2016S containing 48.5% carbohydrates, 4% fat and 0% cholesterol with energy density of 3.0 kcal/g; Harlan Laboratories) was fed to the corresponding groups for 15 weeks. The mice were weighed once a week. Altogether seven ApoE3-TR mice, nine ApoE4-TR mice and seven WT mice died during the study. The overall mortality throughout the study period was around 10% and there were no differences in the mortality rate amongst the different treatment groups. In addition, a separate cohort of 4-month-old ApoE4-TR mice (*n* = 8) and C57BL/6j mice (*n* = 8) was subjected to ischemia.

### Ischemia surgery

At the age of 16 or 17 months, the mice were anesthetized with 5% isoflurane for induction and 2% isoflurane for maintenance (70% N_2_O/30% O_2_). Rectal temperature was maintained at 36.5 ± 0.5°C using a thermostatically controlled rectal probe connected to a homoeothermic blanket during surgery (PanLab, Harvard Apparatus, Barcelona, Spain). The middle cerebral artery (MCA) was permanently coagulated as described earlier [15]. In brief, a small incision was made between the ear and the eye. The temporalis muscle was retracted and the skull exposed. A small hole, about 1 mm in diameter, was drilled on the temporal bone above the MCA at the level of inferior cerebral vein. The dura was carefully removed and the MCA cauterized permanently using a thermocoagulator (Bovie Medical Corporation, Clearwater, FL, USA). After the procedure, the muscle was replaced and the skin wound sutured. Sham-operated mice underwent the same procedure except for coagulation of MCA. After recovery from the anesthesia, the animals were returned to their individual home cages. The mice were terminally anesthetized with 250 mg/kg Avertin (2,2,2-tribromoethanol in tertiary amyl alcohol) and perfused either 3 days or 10 days post surgery. The predetermined humane endpoint was set as follows: the mice that lost more than 10% of their body weight and did not groom or move normally were to be sacrificed before the end of the study. None of the mice in the study met these criteria.

### Physiological parameters

A separate cohort of animals from each genotype (*n* = 5 to 6) was used to measure physiological parameters immediately after MCA occlusion using blood samples drawn from the saphenous vein. Separate cohorts were used to ensure the blood withdrawal would not affect the outcome of ischemia. Glucose levels were measured using the Freestyle blood glucose monitoring system (Abbott, Alameda, CA, USA). The partial pressure of carbon dioxide and oxygen and the pH were measured using an iSTAT analyzer (Abbott, Abbott Park, IL, USA).

### Cholesterol measurement

For serum cholesterol measurement, blood samples were collected via cardiac puncture 10 days after the surgery. Total cholesterol was quantified from serum samples using a cholesterol measurement kit according to the manufacturer’s instructions (Cayman Chemicals, Ann Arbor, MI, USA). Data were collected using a Wallace 1420 workstation (Perkin Elmer, Waltham, MA, USA). The serum cholesterol levels were measured from total of 73 ischemic mice 10 days post surgery in the following treatment groups: WT sham mice on normal chow (WTND + sham, *n* = 7), WT ischemic mice on normal chow (WTND + Isch, *n* = 15) and on HF diet (WTHF + Isch, *n* = 7), ApoE3-TR sham mice on normal chow (E3ND + sham, *n* = 5) and ischemic mice on normal chow (E3ND + Isch, *n* = 7) and HF diet (E3HF + Isch, *n* = 8), and ApoE4-TR sham mice on normal chow (E4ND + sham, *n* = 10) and ischemic mice on normal chow (E4ND + Isch, *n* = 9) and HF diet (E4HF + Isch, *n* = 5).

### Magnetic resonance imaging

The total infarction volume was imaged by magnetic resonance imaging (MRI) at 3 days post ischemia using a vertical 9.4 T Oxford NMR 400 magnet (Oxford Instrument PLC, Abingdon, UK). Mice were anesthetized with isoflurane as described above. A quadrature volume coil was used for transmission and reception. Scout images were obtained to locate the area of the brain and axial sections were acquired right from the start of olfactory bulbs. Multislice T2 weighted images (repetition time 3,000 ms, echo time 40 ms, matrix size 128×256, field of view 19.2×19.2 mm^2^, slice thickness 0.8 mm and number of slices 12) were obtained with double spin-echo sequence with adiabatic refocusing pulse. The obtained images were analyzed by defining the region of interest using in-house made software (Aedes) under the Matlab environment (Math-works, Natick, MA, USA). The total infarction volume and the volumes of left and right healthy hemispheres were calculated from 12 consecutive slices. The relative percentage of infarction volume was calculated using the following formula as described previously [16]:

Infarct volume = (volume of left hemisphere – (volume of right hemisphere – measured infarct volume))/volume of left hemisphere

Analysis was performed blinded to the study groups. The infarction volume was measured from a total of 57 ischemic mice 3 days post surgery in the following treatment groups: WTND, *n* = 15; WTHF, *n* = 8; E3ND, *n* =8; E3HF, *n* = 9; E4ND, *n* = 9; and E4HF, *n* = 8.

### Adhesive removal test

Sensorimotor deficits were evaluated using an adhesive removal test [17]. Briefly, the mouse was first taken from the home cage and adhesive patches of 6.5 mm diameter (Bel-Art Products, Wayne, NJ, USA) were placed on both front paws in random order. The mouse was then placed into a cubicle box and the latencies to sensing and removal of patches from both paws were recorded. Each mouse underwent three trials and the time limit for removing the tape was set to 120 seconds. Animals were tested 3 days prior to and 3 and 7 days after ischemia. Testing and evaluation were carried out by the same person in blinded fashion. Total of 87 animals were evaluated in following groups: WTND + sham, *n* = 8; WTND + Isch, *n* = 15; WTHF + Isch, *n* = 10; E3ND + sham, *n* = 6; E3ND + Isch, *n* = 8; E3HF + Isch, *n* = 9; E4ND + Sham, *n* = 10; E4ND + Isch, *n* = 13; and E4HF + Isch, *n* = 8.

### Cytokine measurement

Cytokines were measured from plasma samples. Blood was collected by cardiac puncture 3 days post ischemia using sodium citrate as an anticoagulant. The levels of IL-6, IL-10, monocyte chemotactic protein-1 (MCP-1), TNF, IFNγ and IL-12p70 were measured using the Cytometric Bead Array and Mouse Inflammation Kit (BD Biosciences, San Jose, CA, USA) according to the manufacturer’s instructions. The samples were run on a FACS Calibur flow cytometer (BD Biosciences) and analyzed using FCAP Array software (Soft Flow Hungary Ltd, Pecs, Hungary). The concentration of MCP-1 was further analyzed by the MCP-1 ELISA kit (Thermo Fischer Scientific, Rockford, IL, USA) because of its higher sensitivity. The plasma cytokine levels were measured from ischemic mice at 3 days post surgery in WTND (*n* = 12), WTHF (*n* = 11), E3ND (*n* = 6), E3HF (*n* = 6), E4ND (*n* = 10) and E4HF (*n* = 10).

### Immunohistochemistry

The brain gliosis was analyzed using ionized calcium-binding adapter molecule-1 (Iba-1) and glial fibrillary acid protein (GFAP) antibodies detecting microglia along with infiltrating macrophages and astrocytes, respectively, by immunohistochemistry at 3 and 10 days post surgery. Neurogenesis was assessed by doublecortin (DCX) antibody detecting immature neurons at 10 days post surgery. In addition, cyclooxygenase-2 (COX-2) immunoreactivity at 3 days post surgery was also accessed. Briefly, the mice were transcardially perfused with heparinized (2,500 IU/l) saline and the brains were post-fixed in 4% paraformaldehyde overnight followed by cryoprotection in 30% sucrose for 3 days. The brains were frozen on liquid nitrogen and cut on 20 μm thick serial coronal sections using a cryostat (Leica Microsystems GmH, Wetzlar, Germany). The sections were reacted against primary antibodies (Iba-1, 1:250 dilution; Wako Chemicals GmbH, Neuss, Germany; GFAP, 1:500 dilution; DAKO, Glostrup, Denmark; DCX, 1:200 dilution; Cell Signaling, Danvers, MA, USA; COX-2, 1:500 dilutio;, Cayman Chemicals) overnight at room temperature. After washing with PBS containing 0.5% Tween20 (Sigma-Aldrich, St. Louis, MO, USA), the sections were incubated against biotinylated secondary antibody (1:200 dilution; Vector, Burlingame, CA, USA) for 2 hours and subsequently reacted against avidin–biotin complex reagent (Vector) according to the manufacturer’s instructions. Alternatively, Alexa 568 conjugated secondary antibody (1:200 dilution; Invitrogen, Eugene, OR, USA) was used. The bound immunoreactivities were visualized by development with nickel-enhanced 3,3′-diaminobenzidine.

For quantification of GFAP, Iba-1 and COX-2 immunoreactivities in the peri-ischemia area, a 718 μm × 532 μm cortical area immediately adjacent to the infarct border was imaged using an AX70 microscope (Olympus Corporation, Tokyo, Japan) on 10× magnification with an attached digital camera (Color View 12 or F-View; Soft Imaging System, Muenster, Germany) running Analysis Software (Soft Imaging System). In addition, a corresponding area on the contralateral hemisphere was similarly analyzed. A total of six consecutive sections in 400 μm intervals starting from the beginning of the ischemic lesion were imaged. DCX immunoreactivity was quantified from six consecutive sections in 400 μm intervals from the subventricular area of 718 μm × 532 μm in size adjacent to the wall of the lateral ventricle in both hemispheres. Immunoreactive areas were quantified blinded to the study groups using ImagePro Plus Software (Media Cybernetics, Rockville, MD, USA) at a predefined range and represented as the percentage of positively stained immunoreactive area.

GFAP immunoreactivity was quantified from a total of 43 ischemic mice in the following groups at 3 days post stroke: WTND, *n* = 8; WTHF, *n* = 8; E3ND, *n* = 6; E3HF, *n* = 6; E4ND, *n* = 8; and E4HF, *n* = 7. Similarly, Iba-1 immunoreactivity was quantified from a total of 42 ischemic mice at 3 days post stroke: WTND, *n* = 8; WTHF, *n* = 7; E3ND, *n* = 6; E3HF, *n* = 6; E4ND, *n* = 7; and E4HF, *n* = 8. COX-2 immunoreactivity was quantified from total of 41 ischemic mice at 3 days post stroke: WTND, *n* = 8; WTHF, *n* = 7; E3ND, *n* = 6; E3HF, *n* = 6; E4ND, *n* = 7; and E4HF, *n* = 7. For detecting astrogliosis and microgliosis at 10 days post stroke, the same groups were stained against GFAP and Iba-1 antibodies with six mice in each. To elucidate the effect of ApoE in ischemia-induced neurogenesis in the context of a HF diet, DCX immunoreactivity was quantified from all study groups (total of 55 mice) at 10 days post stroke: WTND + Sham, *n* = 7; WTND + Isch, *n* = 6; WTHF + Isch, *n* = 5; E3ND + Sham, *n* = 5; E3ND + Isch, *n* = 6; E3HF + Isch, *n* = 6; E4ND + Sham, *n* = 9; E4ND + Isch, *n* = 6; and E4HF + Isch, *n* = 5.

### Assessment of atherosclerotic lesions

Cross-sections of the aortic valve were taken for determination of the development of atherosclerotic lesions. After perfusion, the heart was removed and fixed in 4% paraformaldehyde. The apex of the heart was removed and base of the heart was cast in paraffin. Five-micrometer sections were collected from the starting point of aortic valves toward the base of the heart and H & E staining was performed on every fifth section (total of six to eight sections) for the observation of possible lesions.

### Statistical analysis and exclusion criteria

Statistical analysis was carried out using SPSS 19 (IBM SPSS Inc., Chicago, IL, USA) software. The effect of treat-ment and genotype as dependent variables was analyzed by two-way analysis of variance followed by the Sidak *post-hoc* test. Logarithmic transformation was applied to normalize the data when appropriate. *P* < 0.05 was considered statistically significant. Exclusion criteria were set prior to the start of the experiments. Statistical outliers as analyzed by Graph Pad QuickCalcs, mice with bleeding during the surgery and hemorrhage visible as black spots in the ischemic hemisphere in MRI images were excluded from the dataset. Altogether 13 mice were excluded on this basis: WTND + Isch, *n* = 2; WTHF + Isch, *n* = 3; E3ND + Isch, *n* = 2; E4ND + Isch, *n* = 3; and E4HF + Isch, *n* = 3. Hemorrhage transformation as visible in MRI images was observed only in one mouse of the ApoE3-TR group fed with ND. The data are presented as mean ± standard error of the mean.

## Results

### High-fat diet increases serum total cholesterol levels in both ApoE3-TR and ApoE4-TR mice

**Figure 1:**
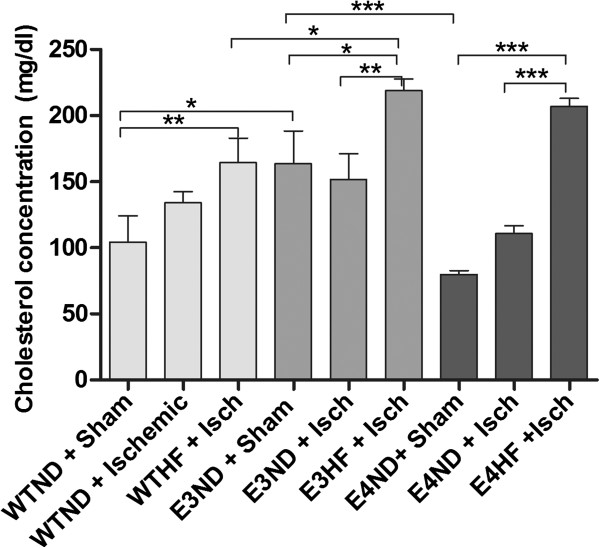
**High-fat diet increases total serum cholesterol in aged ApoE3-TR and ApoE4-TR mice to similar levels.** Serum cholesterol levels were measured 10 days post stroke. ApoE3-TR and ApoE4-TR mice on a high-fat (HF) diet showed similar cholesterol levels. ApoE, apolipoprotein E; E3, E4 and WT, ApoE3-TR, ApoE4-TR and control mice; ND, normal diet; Sham and Isch, sham-operated and ischemic mice; TR, targeted replacement. Data expressed as mean ± standard error of the mean. WTND + sham, *n* = 7; WTND + Isch, *n* = 15; WTHF + Isch, *n* = 7; E3ND + sham, *n* = 5; E3ND + Isch, *n* = 7; E3HF + Isch, *n* = 8; E4ND + sham, *n* = 10; E4ND + Isch, *n* = 9; E4HF + Isch, *n* = 5. * p < 0.05, ** p < 0.01 and *** p < 0.001.

Monitoring the weight of the mice revealed 20% greater body weight in ApoE3-TR mice (38.1 ± 6.0 g) compared with WT (30.5 ± 3.1 g) and ApoE4-TR (31.4 ± 3.4 g) mice prior to the diet *(P* < 0.001) and the difference lasted throughout the HF diet period. The body weight of WT and ApoE4-TR mice was very similar. The weight of all mice reached their maximum 13 weeks after onset of the HF diet and remained at the same level thereafter. At the end of the HF diet period, the average weight (mean ± standard error of the mean) of WT mice was 40.7 ± 2.10 g, the average weight of ApoE3-TR mice was 46.2 ± 2.55 g, and the average weight of ApoE4-TR mice was 39.3 ± 1.49 g. The HF diet increased the serum cholesterol levels significantly to a similar extent in both ApoE3-TR and ApoE4-TR mice, but not in WT mice in which the serum cholesterol levels remained significantly lower compared with ApoE3-TR mice (Figure 1). Interestingly, the serum cholesterol levels in sham-operated ApoE3-TR mice fed with normal chow were significantly higher compared with sham-operated ApoE4-TR mice on normal chow.

### ApoE4-TR mice fed with a high-fat diet exhibit significant sensorimotor deficits upon MCA occlusion independent of the lesion size

**Figure 2:**
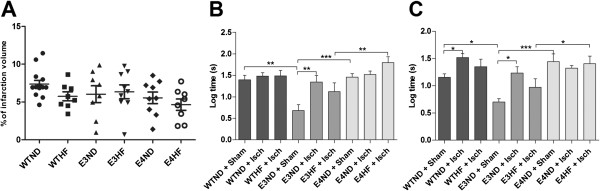
**Effect of apolipoprotein E isoforms alone or combined with a western diet on infarct size.** A long-term high-fat (HF) diet results in clear sensorimotor deficits in ApoE4-TR mice 3 days after permanent middle cerebral artery occlusion without effect on the lesion size. **(A)** Infarct lesions were quantified from magnetic resonance images taken 72 hours post ischemia for WTND (*n* = 15), WTHF (*n* = 8), E3ND (*n* = 8), E3HF (*n* = 9), E4ND (*n* = 9) and E4HF (*n* = 8). Sensorimotor deficits were assessed using an adhesive removal test at **(B)** 3 days and **(C)** 7 days post ischemia. ApoE, apolipoprotein E; E3, E4 and WT, ApoE3-TR, ApoE4-TR and control mice; ND, normal diet; Sham and Isch, sham-operated and ischemic mice; TR, targeted replacement; WT, wild type. Data expressed as mean ± standard error of the mean. WTND + Sham, n = 8; WTND + Isch, n = 15; WTHF + Isch, *n* = 10; E3ND + Sham, *n* = 6; E3ND + Isch, *n* = 8; E3HF + Isch, *n* = 9; E4ND + Sham, *n* = 10; E4ND + Isch, *n* = 13; E4HF + Isch, *n* = 8. * p < 0.05, ** p < 0.01 and *** p < 0.001.

Physiological parameters did not differ between the study groups (Table 1). ApoE isoforms alone or in combination with a western diet had no effect on the infarct size (Figure 2A). Our distal pMCAo model produced a small cortical infarct and the data from MRI analysis revealed no differences between the study groups. To detect whether the ApoE4 isoform alone in young mice influenced the lesion size, 4-month-old ApoE4-TR mice and their WT C57BL/6j controls were subjected to pMCAo. There was no difference in the lesion size between the young WT and ApoE4-TR mice (3.6 ± 2.5 and 3.1 ± 1.7, respectively) and did not significantly differ from the lesion size of the aged mice in any of the study groups.

**Table 1:** Physiological parameters immediately after the onset of ischemia

	**pH**	**pCO**_**2**_**(mmHg)**	**pO**_**2**_**(mmHg)**	**Glucose (mmol/l)**
ApoE3-TR	7.3 ± 0.1	38.8 ± 7.4	44.5 ± 4.5	9.1 ± 1.3
ApoE4-TR	7.3 ± 0.1	38.5 ± 8.5	40.5 ± 3.0	7.7 ± 1.5
Wild type	7.3 ± 0.1	38.9 ± 8.3	42.5 ± 4.5	9.6 ± 1.2

ApoE, apolipoprotein E; pCO_2_, partial pressure of carbon dioxide; pO_2_, partial pressure of oxygen; TR, targeted replacement.

Long-term intake of a HF diet resulted in clear sensorimotor deficits in ApoE4-TR mice 3 days after pMCAo (Figure 2B). Interestingly, the ApoE3-TR mice on the ND and not suffering from stroke were faster in removing the tapes compared with sham-operated mice of other genotypes. Stroke alone was not able to induce statistically significant loss in sensorimotor functions in ApoE4-TR mice, whereas in the ApoE3 genotype, the superior sensorimotor functions seen in sham mice were impaired after ischemia to the level of other genotypes. On the other hand, ApoE4-TR mice fed on a HF diet showed significantly aggravated ischemia-induced deficits com-pared with ApoE3-TR mice on the same diet (Figure 2B). The sensorimotor deficits caused by pMCAo persisted until day 7 post ischemia, and at this time point the ischemia-induced deficits became apparent in WT mice (Figure 2C).

### ApoE3-TR and ApoE4-TR mice fed with a high-fat diet show increased GFAP immunoreactivity upon permanent middle cerebral artery occlusion

**Figure 3:**
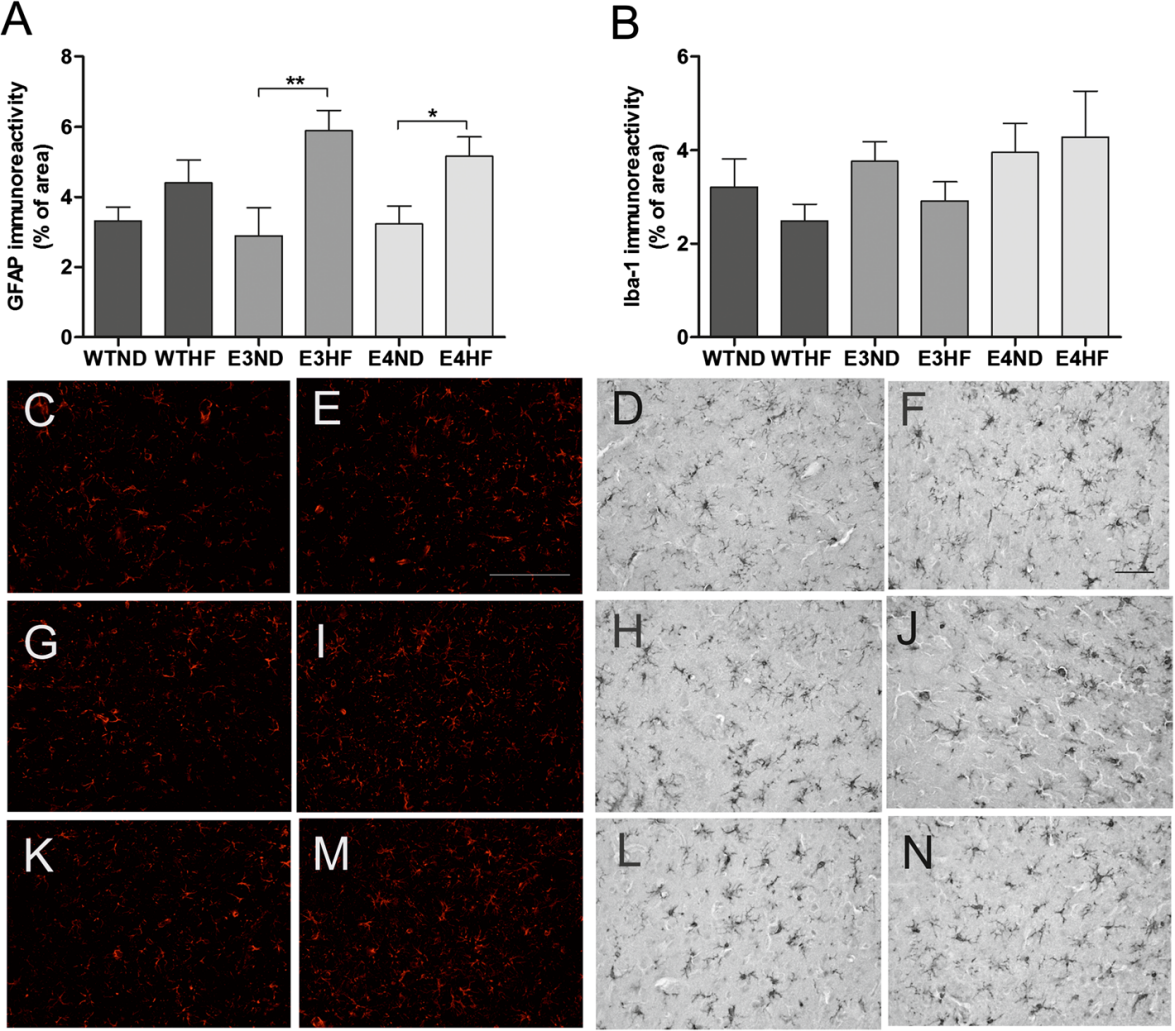
**Ischemia-induced brain astrogliosis is altered upon receiving a high-fat diet. (A)** Glial fibrillary acidic protein (GFAP) and **(B)** Iba-1 immunoreactivities were quantified from the peri-ischemic area at 3 days post stroke. Example images of GFAP and Iba-1 staining in the peri-ischemic region of wild-type (WT) mice on **(C**, **D)** a normal diet (ND) and **(E**, **F)** a high-fat (HF) diet, of ApoE3-TR mice on **(G**, **H)** normal chow and **(I**, **J)** a HF diet, and of ApoE4-TR mice on **(K**, **L)** normal chow and **(M**, **N)** a HF diet, respectively. ApoE, apolipoprotein E; E3, E4 and WT, ApoE3-TR, ApoE4-TR and control mice; TR, targeted replacement. Scale bar = 50 μm. Data expressed as mean ± standard error of the mean. For quantification of GFAP immunoreactivity: WTND, *n* = 8; WTHF, *n* = 8; E3ND, *n* = 6; E3HF, *n* = 6; E4ND, *n* = 8; E4HF, *n* = 7. For quantification of Iba-1 immunoreactivity: WTND, *n* = 8; WTHF, *n* = 7; E3ND, *n* = 6; E3HF, *n* = 6; E4ND, *n* = 7; E4HF, *n* = 8. * p < 0.05 and ** p < 0.01.

Brain glial activation was analyzed by immunostaining the brain sections against Iba-1 and GFAP antibodies. Quantification of immunoreactivities revealed that ischemia caused a significant upregulation in both glial markers in the peri-ischemic area when compared with the contralateral side in all treatment groups at 3 and 10 days post stroke (data not shown). Ischemia induced GFAP upregulation in the peri-ischemic area to a similar extent in WT, ApoE3-TR and ApoE4-TR mice kept on a ND at 3 days post ischemia (Figure 3A). The HF diet actuated a further, significant increase in GFAP immuno-reactivity in the peri-ischemic area in ApoE3-TR and ApoE4-TR mice but not in WT mice at 3 days post ischemia. The extent of GFAP immunoreactivity in the peri-ischemic area between ApoE3-TR and ApoE4-TR mice fed with a HF diet was similar (Figure 3A). At 10 days post ischemia, the HF diet-accompanied upregulation in astrocytic activation was diminished (data not shown).

Ischemia induced a significant increase in Iba-1 immunoreactivity in the peri-ischemic area compared with the contralateral side in all treatment groups at 3 days (Figure 3B) and 10 days post stroke (data not shown). There were no significant differences in the extent in Iba-1 immunoreactivity in the peri-ischemic area between the genotypes regardless of their dietary intake at either 3 or 10 days after ischemia. Figure 3C to N depicts typical example images of the GFAP and Iba-1 im-munoreactivities in the peri-ischemic area in WTND, WTHF, E3ND, E3HF, E4ND and E4HF, respectively.

### High-fat diet increases ischemia-induced COX-2 activation in the peri-ischemic area in ApoE4-TR mice but not in wild-type or ApoE3-TR mice

**Figure 4:**
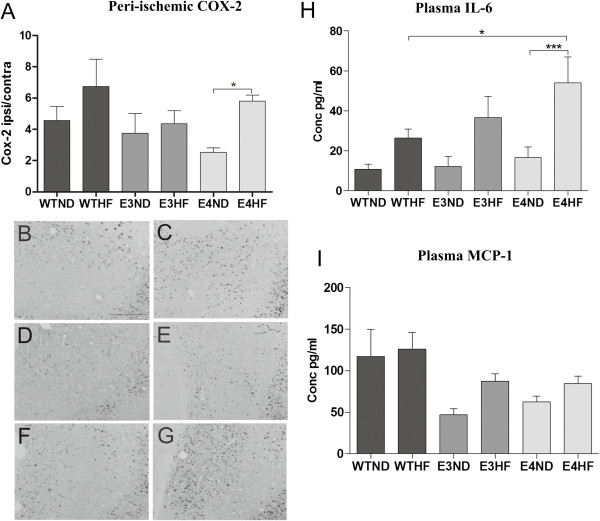
**High-fat diet aggravates ischemia-induced cyclooxygenase-2 immunoreactivity and plasma IL-6 levels in ApoE4-TR mice.** Cyclooxygenase-2 (COX-2) immunoreactivity was quantified 3 days post stroke. **(A)** A high-fat (HF) diet increased the extent of COX-2 immunoreactivity in ApoE4-TR mice but failed to do so in wild-type (WT) or ApoE3-TR mice. * p < 0.05. Typical example images of the COX-2 immunoreactivity in the peri-ischemic area in: **(B)** WTND, **(C)** WTHF, **(D)** E3ND, **(E)** E3HF, **(F)** E4ND and **(G)** E4HF. ApoE, apolipoprotein E; E3, E4 and WT, ApoE3-TR, ApoE4-TR and control mice; ND, normal diet; TR, targeted replacement. Scale bar = 50 μm. Values presented as a ratio between ipsilateral and contralateral hemispheres. Data expressed as mean ± standard error of the mean (SEM). WTND, *n* = 8; WTHF, *n* = 7; E3ND, *n* = 6; E3HF, *n* = 6; E4ND, *n* = 7; E4HF, *n* = 7. Plasma **(H)** IL-6 and **(I)** monocyte chemoattractant protein-1 (MCP-1) levels were measured 3 days after ischemia. Data expressed as mean ± SEM. WTND, *n* = 12; WTHF, *n* = 11; E3ND, *n* = 6; E3HF, *n* = 6; E4ND, *n* = 10; E4HF, *n* = 10. * p < 0.05 and *** p < 0.001.

Because microglia and astrocytes are not the only mediators in the ischemia-induced inflammation, we assessed whether the ischemia-induced increase in COX-2 activation in the peri-ischemic area was altered upon the ApoE genotype and HF exposure. Quantification of COX-2 immunoreactivity revealed an ischemia-induced upregulation in COX-2 in the peri-ischemic area at 3 days post stroke in all treatment groups except in ApoE3-TR mice on normal chow. A HF diet increased significantly the extent of COX-2 immunoreactivity in ApoE4-TR mice but failed to do so in WT or in ApoE3-TR mice (Figure 4A). Figure 4B to G depicts typical example images of the COX-2 immunoreactivity in the peri-ischemic area in WTND, WTHF, E3ND, E3HF, E4ND and E4HF, respectively.

### ApoE4-TR mice fed a high-fat diet increase plasma IL-6 levels upon permanent middle cerebral artery occlusion

Of various cytokines tested (IL-6, IL-10, MCP-1, TNF, IFNγ and IL-12p70), only IL-6 and MCP-1 were found within detectable limits in the plasma samples analyzed at 3 days post ischemia. Cytometric bead array revealed a significant induction in plasma IL-6 in ApoE4-TR mice fed with a HF diet mice compared with their genotype counterparts on normal chow (Figure 4H). The plasma levels of IL-6 in ApoE4-TR mice fed with a HF diet were significantly higher compared with the levels in WT mice on a HF diet. ELISA of MCP-1 did not reveal any significant differences between the study groups (Figure 4I).

### ApoE3-TR and ApoE4-TR mice do not develop atherosclerotic lesions in aortic valves

**Figure 5:**
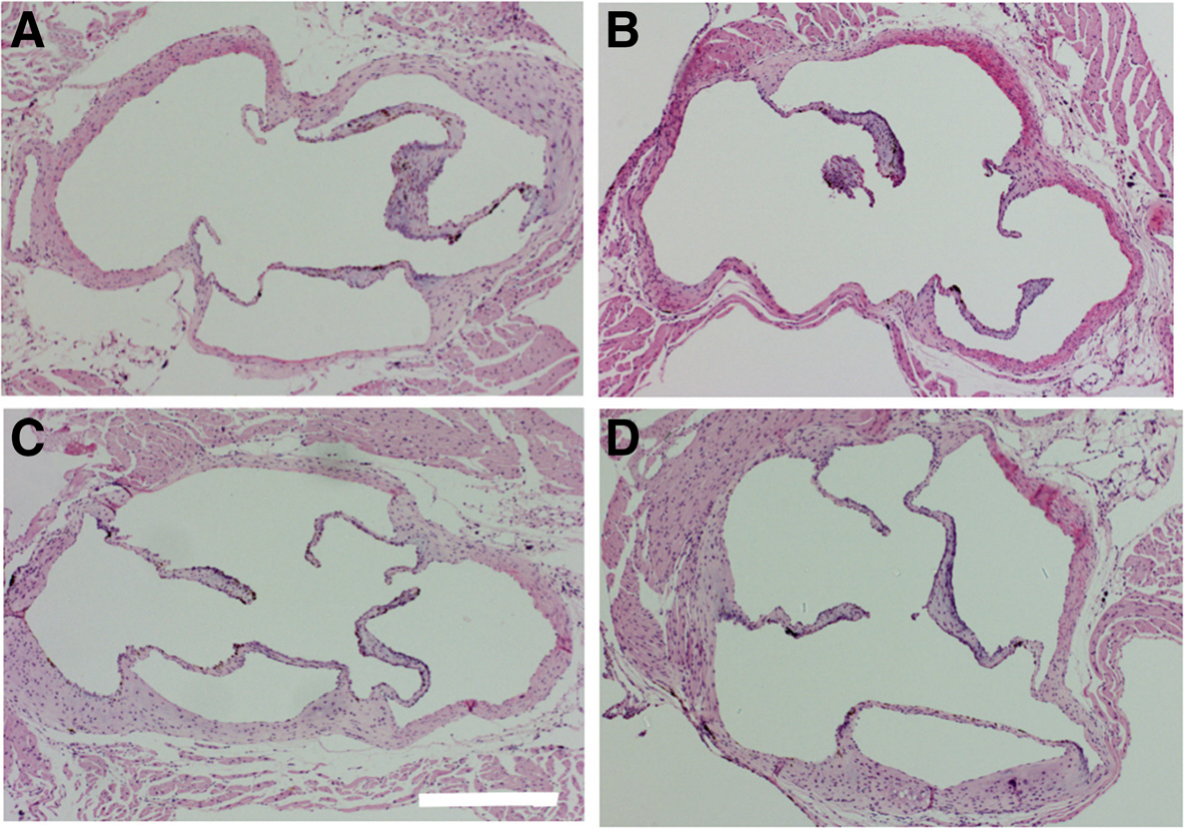
**High-fat diet did not induce atherosclerotic lesions in the aortic valve.** A high-fat (HF) diet did not induce atherosclerotic lesions as evidenced by H & E stain of the aortic valve. Fatty streak plaques were absent in all three strains: **(A)** ApoE4-TR fed a HF diet, **(B)** ApoE3-TR fed a HF diet and **(C)** wild-type (WT) mice fed a HF diet. ApoE, apolipoprotein E; TR, targeted replacement. **(D)** Typical example image showing a WT mouse on normal diet. Representative photomicrographs are shown with original magnification of ×4. Scale bar = 500 μm.

The aortic valve surface from all strains, ApoE4-TR, ApoE3-TR and WT mice, on a HF diet appeared to have an intact covering with smooth endothelial cell layer as demonstrated by H & E staining (Figure 5A,B,C, respectively) and resembled the aortic valve surface of WT mice fed with normal chow (Figure 5D). None of the study groups showed foam cell or fatty plaque formation on the endocardial surface of the valve leaflets, indicating that the intake of the HF diet used in this study was unable to induce atherosclerosis in WT, ApoE3-TR and ApoE4-TR mice. The only abnormality found in the aortic valves was a small inclusion of inflammatory cells underneath the endothelial cell layer in one of the ApoE4-TR mice fed with a HF diet and thus this finding was regarded as insignificant.

### Human apolipoprotein E isoforms alter the ischemia-induced induction of neurogenesis

**Figure 6:**
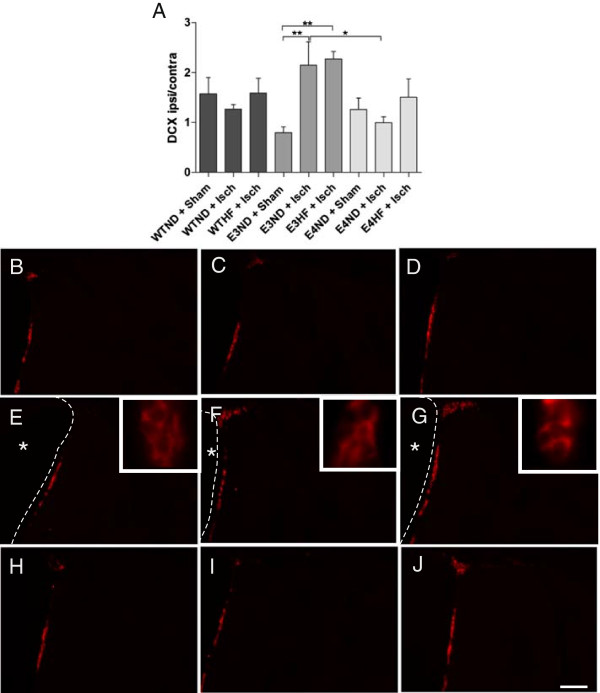
**ApoE3-TR mice have increased ability to upregulate neurogenesis upon ischemic injury.** Newly born neurons were visualized by doublecortin (DCX) staining at 10 days post ischemia. **(A)** DCX-positive cells were quantified from the subventricular zone of both hemispheres and the values are presented as a ratio between ipsilateral and contralateral hemispheres. * p < 0.05 and ** p < 0.01. Typical example images of DCX immunoreactivity in the subventricular zone in the ipsilateral hemisphere of: **(B)** sham-operated wild-type (WT) mice on a normal diet (ND, *n* = 7), **(C)** ischemic WT mice on a ND (*n* = 6) and **(D)** on a high-fat (HF) diet (*n* = 5), **(E)** sham-operated ApoE3-TR mice on normal diet (*n* = 5), **(F)** ischemic ApoE3-TR mice on normal diet (*n* = 6) and **(G)** on a HF diet (*n* = 6), **(H)** sham-operated ApoE4-TR mice on normal diet (*n* = 9), and **(I)** ischemic ApoE4-TR mice on normal diet (*n* = 6) and **(J)** on a HF diet (*n* = 5). Dashed line, lining of the ventricular wall; asterisks, lateral ventricle on the ipsilateral hemisphere. ApoE, apolipoprotein E; E3, E4 and WT, ApoE3-TR, ApoE4-TR and control mice; Sham and Isch, sham-operated and ischemic mice; TR, targeted replacement. Data expressed as mean ± standard error of the mean. Scale bar = 100 μm.

Neurogenesis is one of the mechanisms associated with improved recovery upon ischemic insults in many studies. The ApoE4 allele has been shown to dampen neurogenesis [18,19], so we sought to determine whether the poorer outcome in the adhesive removal test in ApoE4-TR mice on a HF diet is associated with a decline in ischemia-induced neurogenesis. The amount of newly born neurons was very limited due to the old age of the animals. Quantification revealed that ischemia failed to induce significant neurogenesis in WT and ApoE4-TR mice, but a significant ischemia-induced induction in the amount of newly born neurons was evident in ApoE3-TR mice fed a ND (Figure 6A). The number of newly born neurons in ApoE3-TR mice on normal chow was significantly higher compared with ApoE4-TR mice fed with the same diet. The HF diet did not have any additional impact on ischemia-induced endogenous neurogenesis. Figure 6B to J depicts typical example images of the DCX immunoreactivity in the subventricular zone in WTND + Sham, WTND + Isch, WTHF + Isch, E3ND + Sham, E3ND + Isch, E3HF + Isch, E4ND + Sham, E4ND + Isch and E4HF + Isch.

## Discussion

APOE*4 has been shown to augment injury and worsen outcome in various models of neurodegeneration, including cerebral hemorrhage [20], traumatic brain injury (TBI) [21], stroke [8,11,22] and global brain ischemia [23], and has been suggested to be a significant risk factor for cognitive impairment in the early phase after stroke [24]. We assessed the contribution of ApoE4 to the outcome of focal brain ischemia in aged mice maintained on a HF diet and show for the first time that ApoE4 together with long-term intake of a HF diet containing 5.3 times more fat and having 1.5 times higher energy density compared with normal chow leads to significant sensorimotor deficits as analyzed by an adhesive removal test. These exacerbated deficits were not due to altered lesion size but were associated with altered peri-ischemic astrogliosis and increase in plasma IL-6 levels.

Intriguingly, ApoE3-TR mice fed with a ND and not suffering from stroke were superior in the adhesive removal test, especially at 3 days post ischemia. The reason for this observation is not known. Neurons in ApoE3-TR mice have been shown to exhibit more complex branching pattern of dendrites compared with ApoE4-TR or WT C57BL-mice and the mice display enhanced excitatory activity that is not reduced by aging in contrast to ApoE4-TR and C57BL mice [25]. Likewise, the ApoE4-TR mice show the lowest levels of excitatory synaptic activity [25], reduced long-term potentiation [26] and poorer outcome in tasks measuring spatial memory [27]. In fact, the ApoE4 allele in older people has been shown to be associated with a two fold increase in the rate of motor decline [28].

In the current study, the deficits in the adhesive removal test became apparent in ApoE4-TR mice on a HF diet compared with ApoE3-TR mice under the same diet, and the deficits persisted until 7 days post injury. In contrast, even though WT mice displayed sensorimotor deficits at 7 days post stroke and ApoE3-TR mice at 3 and 7 days post stroke, a HF diet did not aggravate or result in statistically significant sensorimotor defects after pMCAo in any of the groups compared with their genotype controls on normal chow. The lesion resulting from this ischemia model is relatively small and strictly cortical and thus the deficits in adhesive removal test were fairly moderate, causing a limitation in this study. Even though aged ApoE4-TR mice fed with a HF diet showed pronounced sensorimotor deficits, there were no differences in the size of the ischemic lesion. This indicates that the motor deficits were independent of the magnitude of neuronal loss in the cortex upon ischemic insult, and the expression of human APOE*4 in combination with a HF diet is likely to interfere with recovery or compensation mechanisms of the lost neuronal functions after cortical brain infarction.

Indeed, other studies have highlighted the impact of a HF diet on the behavioral impairment [29]. A HF diet has been shown to be related to decreased levels of brain-derived neurotrophic factor, which is crucial for synaptic plasticity and learning [29]. In addition, a diet containing 60% of fat alone has been shown to impair learning and memory in 12-month-old C57BL mice [30]. Even though a previous study has shown an increase in the lesion size in young ApoE4 knock-in mice maintained on a ND [22], others have reported findings similar to ours in other models of neurodegeneration. ApoE4 mice have been shown to have poor functional outcome independent of the lesion size in models of hemorrhage [20] and TBI [21], and in fact the lesion size in immature ApoE4 transgenic mice upon TBI was significantly decreased [21]. A previous study showing an increase in the lesion size in ApoE4-TR mice used a different permanent focal ischemia model, which resulted in a larger lesion compared with the current study [22], at least partially explaining the different outcome. To dissect out whether age of the mice used in the current study may have contributed to the outcome, we subjected 4-month-old ApoE4 mice and C57BL/6j mice to pMCAo. In agreement with our data obtained from the aged animals, the size of the lesion in ApoE4-TR mice did not differ from their WT controls or any of the aged study groups used in the current experimental setup.

The effect of age of ApoE4 mice in the lesion size is indeed of interest. Aged mice have been shown to have altered brain gliosis [9] and C57BL mice at the age of 18 to 19 months display increased vulnerability to ischemic damage following transient MCA occlusion [31]. To better meet the pathological features of a typical human stroke patient in the current study, we utilized aged, 16-month-old mice corresponding to an aged patient of increased risk of stroke, fed with a chow resembling a typical unhealthy human cuisine in western countries with the expression of human ApoE isoforms. We also assessed sensorimotor functions, which can be seen as a more relevant outcome measure than the infarct size. Indeed, measurement of functional outcomes and the use of clinically more relevant, aged animal models have been recognized to be of utmost importance in preclinical stroke research [2].

pMCAo triggers astrocytic and microglial activation in the peri-ischemic area. We found that this induction was similar in all genotypes. Instead, a HF diet significantly aggravated the induction in GFAP immunoreactivity in the peri-ischemic region specifically in ApoE3-TR and ApoE4-TR mice but not in WT mice at 3 days post stroke. Astrocytes have been suggested to confer neuroprotection in stroke and other models of CNS damage [32-34], but may also release neurotoxic molecules and even glutamate contributing to neuronal damage upon stroke [35]. Our results point out that dietary intake influences stroke-induced astrocytic activation in the context of human ApoE3 and ApoE4 isoforms. Indeed, a previous paper has reported increased GFAP immunoreactivity in cerebral cortex on a high-cholesterol diet [36] in naive mice.

The fact that Iba-1 immunoreactivity in the peri-ischemic area was significantly increased upon stroke in all genotypes independent of their dietary intake suggest that brain microgliosis as analyzed by Iba-1 immunoreactivity does not contribute to worsened outcome in the ApoE4-TR mice fed with a HF diet. An earlier study shows that microgliosis is not altered in ApoE4-TR mice upon TBI [21]. Since microglia and astrocytes are not the only inflammatory mediators, upon ischemic insult, we determined the peri-ischemic COX-2 immunoreactivity at 3 days post stroke. While a HF diet failed to induce significant increase in the peri-ischemic COX-2 immunoreactivity in WT or ApoE3-TR mice, ischemic ApoE4-TR mice fed a HF diet exhibited significantly increased levels of COX-2 immunoreactivity in the peri-ischemic area. Previous literature has shown that COX-2 aggravates neuronal injury in early stages after stroke [37] and a HF diet increases COX-2 in the cerebral cortex in naive rats [38]. In relation to these findings, our results suggest that the ApoE genotype in the context of dietary intake influences the capacity of the brain to cope against ischemic damage. Even though there is a body of evidence that microgliosis/recruitment of peripheral macrophages is a significant determinant of stroke outcome, our results point towards the importance of peripheral inflammation such as induction of IL-6 or CNS inflammation attributed by COX-2 immunoreactivity as possible contributors to the functional outcome after stroke. Indeed, co-morbid conditions aggravating the outcome after ischemia entail a peripheral inflammatory response including plasma IL-6 upregulation [39,40], and high plasma IL-6 levels have been associated with worsened cognitive decline in older people and in APOE*4 carriers [41]. Importantly, whereas in the CNS the increased post-ischemic levels of IL-6 have been suggested to be beneficial, peripheral IL-6 levels correlate well with both stroke severity and poor clinical outcome, suggesting IL-6 to be a marker of harmful inflammation in clinical stroke [42].

Our results are consistent with the hypothesis that ApoE modifies peripheral inflammatory responses in acute and chronic CNS diseases. Peripheral lipopolysaccharide administration increases serum IL-6 levels in ApoE4-TR mice to a higher extent compared with their ApoE3-TR counterparts [8]. These findings have clinical relevance, as similar results of the impact of ApoE4 on peripheral inflammation have been noted also in humans undergoing cardiopulmonary bypass [43,44]. Moreover, patients carrying APOE*3 have a lower incidence of developing severe sepsis in an elective surgical cohort [45] compared with patients lacking APOE*3. Finally, macrophages isolated from individuals carrying APOE*4 show increased NO production during immune activation [46].

In the current study, sham-operated ApoE4-TR mice on normal chow had lower serum cholesterol levels compared with ApoE3-TR mice on same diet. This observation is in contrast to previous report on young mice [47] and thus the old age and gender of the mice in the current study may contribute to this phenomenon. The cholesterol levels in HF diet-fed ApoE3-TR mice were significantly higher compared with their WT controls in agreement with earlier observations in young mice [14]. HF-fed ApoE4-TR mice had similar levels of total serum cholesterol compared with ApoE3-TR mice on the same diet, as similarly described earlier [47]. Despite the increase in serum cholesterol, the HF diet did not lead to formation of atherosclerotic plaques or foam cell accumulation as evidenced by H & E staining of aortic valves. The lack of clear atherosclerotic lesions is in contrast with previous studies showing an increase in atherosclerotic plaque formation in ApoE-TR mice [14,47]. This contrast is most probably explained by difference in the diets, age or gender used between studies. The diet found to cause atherosclerotic plaques [14,47] contains over six times higher levels of cholesterol compared with the diet used in our study. Nevertheless, the fat composition of the Harlan TD.88137 diet used in our study is representative of the western diet linked to the increased risk of cardiovascular diseases in humans.

Neurogenesis is an endogenous repair mechanism serving to amend the lost neuronal functions and has been implicated to be one of the mechanisms associated with improved recovery after brain injury. Indeed, neurogenesis is increased upon stroke [48] as a defense mechanism against neuronal loss. ApoE4 has been implicated to dampen neurogenesis [18] and ApoE isoforms alter the expression of pro-neurogenic genes [19], so we sought to determine whether the sensorimotor deficits in ApoE4-TR mice on a HF diet would accompany reduced neurogenesis. Indeed, our results show that the ischemia-induced level of endogenous neurogenesis in ApoE3-TR mice was higher compared with ApoE4-TR mice and may reflect the impact of a HF diet and specific ApoE background on the ischemia-induced motor deficits. Together with the previously published data [19], our results suggest that ApoE3-TR mice may have increased ability to respond to brain damage compared with ApoE4-TR mice.

## Conclusions

To our knowledge, this is the first study demonstrating the importance of the ApoE genotype to the functional outcome of focal brain ischemia in the context of western dietary intake. Although our results are insufficient to completely explain the observed sensorimotor deficits in ApoE4-TR mice on a HF diet, the increase in plasma IL-6 levels and peri-ischemic COX-2 immunoreactivity in our study suggest the role of peripheral immune responses as well as CNS inflammatory mediators on the outcome after stroke. Furthermore, our study highlights the importance of preclinical stroke models with co-morbid conditions better recapturing clinical human stroke.

## Abbreviations

ApoE: Apolipoprotein; CNS: Central nervous system; COX-2: Cyclooxygenase-2; DCX: Doublecortin; ELISA: Enzyme-linked immunosorbent assay; GFAP: Glial fibrillary acidic protein; H & E: Hematoxylin and eosin; HF: High fat; Iba-1: Ionized calcium-binding adapter molecule 1; IFN: Interferon; IL: Interleukin; MCA: Middle cerebral artery; MCP-1: Monocyte chemotactic protein-1; MRI: Magnetic resonance imaging; ND: Normal diet; PBS: Phosphate-buffered saline; pMCAo: Permanent middle cerebral artery occlusion; TBI: Traumatic brain injury; TNF: Tumor necrosis factor alpha; TR: Targeted replacement; WT: Wild type.

## Competing interest

The authors declare that they have no competing interests.

## Authors’ contributions

HD, TM, TR, ES, SW, and KS carried out the experiments. A-KR provided help in atherosclerosis analysis. PMS provided the animals. HD, TM, PMS and JK planned the study and wrote the manuscript. All authors read and approved the final version of the manuscript.
